# A Text Book of Pathological Anatomy and Pathogenesis

**Published:** 1884-12

**Authors:** 


### Book Reviews.

---

A Text Book of Pathological Anatomy and Pathogenesis, by Ernst Zeigler, Professor of Pathological Anatomy in the University of Tubingen. Translated and edited for English students. By Donald MacAlister, M. A., M. B. Part II. Special Pathological Anatomy, Sec. 1-vii. New York, William Wood & Co. 1884.

If the members of our profession in this country desire to keep pace with the great advances made latterly in pathology, they must necessarily study such works as this which comes from abroad, as there are but few productions of our own which indicate the patient investigation and close research into their details, that is displayed by Prof. Zeigler in this Pathological Anatomy. The demand for it in Germany is not likely to be equaled in North America, from the fact that our professional men look for the most part to the main result, without considering so much the means of securing it, and have put aside the study of the deep-laid principles involved in a thorough comprehension of physical disorders. The second part of this text-book, which is now laid before the public, treats of matters that enter into the every day experience of all classes of practitioners; and the physician as well as the surgeon will find much to enlighten the obscurities of the practical problems of different diseases.

Should any be disposed to accompany Wharton Jones in reopening the question of the primary or secondary relations of the migration of leucocytes to inflammation, he may not find a final and satisfactory solution of all the intricacies growing out of tissue germination and interstitial deposits; but the more tangible changes in the organic structures, requisite for a proper comprehension of the modifications impressed by different forms of disease are well delineated in the various sections of this part of the work.

A prominent place is given to the primitive origin of disorders, saying: "It must also be remembered that many parasites and chiefly the vegetable kinds have the power of multiplication within the blood, so that every drop may contain a multitude of individual organisms. The best example of which are afforded by the anthrax bacillus and spirillium of relapsing fever. In the case of the other, bacterial affections, this process of multiplication within the blood has not yet been demonstrated, though there are many disorders in which brood-colonies are met with in the smaller blood-vessels as in pyæmia. Among animal parasites the Filaria Sanguinis is the only one which occurs in great numbers in human blood. Trichinæ when they do enter the blood stay only a short time in it." Barring the unscientific, though recognized, use of the term anthrax-bacillus in connection with malignant pustule, we must note the tendency at present, amongst pathologists, to account for all diseases upon the bacterial basis; and further developments can only prove the correctness or demonstrate the error of this view. The closing section of the volume upon the liver and pancreas is worthy of special consideration by the practitioner.

This is the September number of Wood's popular library for 1884, and is gotten up in the handsome style characteristic of the publishers.

